# Changes in Staff, Salaries, Departments

**Published:** 1919-12-06

**Authors:** 


### BROMPTON HOSPITAL.

### Changes in Staff, Salaries, Departments.

The Committee of the Brompton Hospital for Consumption report that the number of patients admitted to the hospital since the last court was 665, of whom 132 were transferred to the sanatorium; there were 1,505 new out-patient cases.

The Committee regret the death of Mr. R. H. Horton-Smith, K.C., a valued member of the Committee since 1904 and also that of Sir Philip F. Rose, Bart., a former member of the Committee for many years, and a son of the founder of the hospital. The hospital has also sustained a severe loss in the death of Dr. W. O. Meek, the medical superintendent of the sanatorium, to whose capable management the great benefit derived by the patients at the sanatorium, and the continued success of that branch of the work, are largely due. They have appointed in his place Dr. R. C. Wingfield, from St. Thomas's Hospital.

The majority of the patients received at the hospital for treatment continue to be ex-Service men.

It appears to the Committee desirable that the present vacancy in the post of surgeon should be filled by the appointment of two surgeons instead of one. They recommend that a registrar should be appointed, thus enabling the senior house physician to the hospital also to act as assistant-resident medical officer; the latter formerly had charge of the records of cases. Further, that the superintendent of laboratories should have the assistance of a whole-time assistant, and also that of a part-time assistant to investigate methods in bio-chemistry. They have decided that the *x*-ray department should be open daily, instead of on two days in the week, and have appointed an assistant to the medical officer in charge of the department. That anæsthetics in the dental department should be administered by a visiting anæsthetist, instead of by a member of the resident staff. They have further decided that the time has arrived to establish a cardiographic department, with a director in charge, at an honorarium of fifty guineas per annum. The Committee have decided that the title of resident physician should be conferred on Dr. T. Gywnne Maitland, the present resident medical officer.

The Committee have again considered the question of the salaries paid to the nursing staff, which are now increased to the following scale: Probationers, £20 per annum; staff nurses, £30 per annum, rising to £40; sisters, £50 per annum, rising to £60. In addition, the Committee, to encourage the nursing staff to become policy-holders in the Royal National Pension Fund for Nurses, with which the hospital is federated, have decided to take out, after one year's satisfactory service, instead of three years, as heretofore, similar policies maintained by the hospital in favour of each nurse joining in the scheme. The lectures to nurses, social workers, and others, recently inaugurated on the initiative of Dr. T. Gwynne Maitland, have been welcomed, not only by the nursing staff of the hospital, but by many from other institutions and outside workers, and it is propoesd to continue these.

The Committee have to acknowledge, with many thanks, the receipt of valuable stores from the British Red Cross Society, whom the Committee have also to thank, together with the French Red Cross, for kind assistance in conveying the patients to the sanatorium during the recent railway strike.

